# TIGAR contributes to ischemic tolerance induced by cerebral preconditioning through scavenging of reactive oxygen species and inhibition of apoptosis

**DOI:** 10.1038/srep27096

**Published:** 2016-06-03

**Authors:** Jun-Hao Zhou, Tong-Tong Zhang, Dan-Dan Song, Yun-Fei Xia, Zheng-Hong Qin, Rui Sheng

**Affiliations:** 1 Department of Pharmacology and Laboratory of Aging and Nervous Diseases, Soochow University School of Pharmaceutical Science, Suzhou, China

## Abstract

Previous study showed that TIGAR (TP53-induced glycolysis and apoptosis regulator) protected ischemic brain injury via enhancing pentose phosphate pathway (PPP) flux and preserving mitochondria function. This study was aimed to study the role of TIGAR in cerebral preconditioning. The ischemic preconditioning (IPC) and isoflurane preconditioning (ISO) models were established in primary cultured cortical neurons and in mice. Both IPC and ISO increased TIGAR expression in cortical neurons. Preconditioning might upregulate TIGAR through SP1 transcription factor. Lentivirus mediated knockdown of TIGAR significantly abolished the ischemic tolerance induced by IPC and ISO. ISO also increased TIGAR in mouse cortex and hippocampus and alleviated subsequent brain ischemia-reperfusion injury, while the ischemic tolerance induced by ISO was eliminated with TIGAR knockdown in mouse brain. ISO increased the production of NADPH and glutathione (GSH), and scavenged reactive oxygen species (ROS), while TIGAR knockdown decreased GSH and NADPH production and increased the level of ROS. Supplementation of ROS scavenger NAC and PPP product NADPH effectively rescue the neuronal injury caused by TIGAR deficiency. Notably, TIGAR knockdown inhibited ISO-induced anti-apoptotic effects in cortical neurons. These results suggest that TIGAR participates in the cerebral preconditioning through reduction of ROS and subsequent cell apoptosis.

Stroke is the third common lethal disease in the world with the characteristics of high mortality and disability^1^,^2^. With the aging of population, cardiovascular disease induced stroke is gradually increasing and imposes a heavy social and economic burden on individuals and communities^3^. Thus it is of great importance to study the mechanisms of cerebral vascular disease and to find novel targets for drug development.

Cerebral preconditioning is a temporary, non-lethal injurious procedure below the threshold of damage that makes the brain acquire tolerance against lethal ischemia or hypoxia^4^,^5^,^6^. The therapeutic window of preconditioning will last 1 to 3 days, and it has already showed protective effect in clinics^7^. The ways to induce cerebral preconditioning include non- injurious ischemia (ischemic preconditioning, IPC)^8^, exposure to inhaled anesthetics^9^, hypoxic preconditioning and low doses of endotoxin, etc^10^. Isoflurane is a potent inhalation anesthetic agent that has been used for decades in clinical anesthesia, but the brain can also be preconditioned with isoflurane to reduce neuronal damage in susceptible patients^11^. Isoflurane preconditioning (ISO) participates in both rapid and delayed phases of ischemic tolerance, thereby increasing the survival rate of the neuronal cells^12^,^13^,^14^. Consistently, evidences showed that transient ischemic attacks may precondition humans or animals against stroke and the mechanisms have not been fully understood^15^,^16^,^17^.

TP53-induced glycolysis and apoptosis regulator (TIGAR) was first reported in 2006^18^,^19^. TIGAR functions as a fructose-2,6-bisphosphatase to inhibit glycolysis^20^. When the glycolysis level decreases, the glucose metabolism enters the pentose phosphate pathway (PPP) as compensation^21^. It is known that neurons have a very lower glycolytic rate due to the low 6-phosphofructo-2-kinase/fructose-2,6-bisphosphatase-3 (PFKFB3) activity^22^. Therefore, neurons are unable to enter glycolysis effectively under stressed conditions, while they may preferentially use glucose through the PPP flux to generate GSH and NADPH and to maintain their antioxidant status^23^. The previous results of our lab showed that TIGAR was rapidly upregulated in neurons in response to ischemia/reperfusion. Overexpression of TIGAR reduced ischemic neuronal injury, whereas TIGAR knockdown aggravated ischemic injury^24^. We thus hypothesize that TIGAR might be involved in cerebral preconditioning. In this study, we established IPC and ISO cerebral preconditioning models *in vivo* and *in vitro*. We then investigated whether TIGAR is involved in preconditioning- induced ischemic tolerance.

## Results

### Increases in TIGAR expression in cortical neurons by IPC and ISO

**Figure 1:**
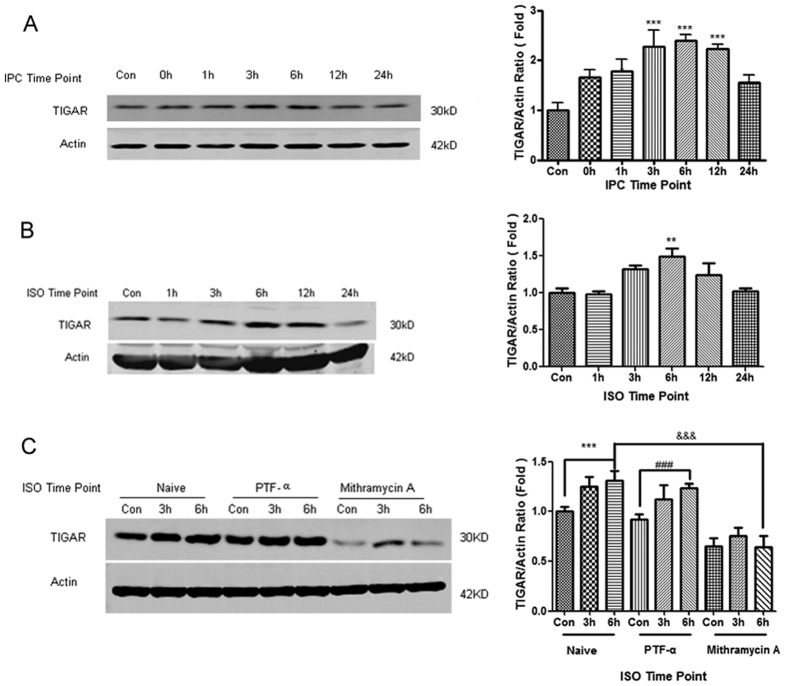
Increases in TIGAR expression in cortical neurons by ischemic preconditioning (IPC) and isoflurane preconditioning (ISO). (**A**) The cortical neurons were exposed to oxygen glucose deprivation (OGD) for 30 min to induce IPC. (**B**) The neurons were exposed to 2% isoflurane for 30 min to induce ISO. (**C**) The neurons were pretreated with PTF-α 10 μM or mithramycin A 300 nM 24 h before ISO. The cells were harvested at the indicated time points after IPC or ISO and The protein levels of TIGAR were detected with Western blot analysis. β-actin levels were used as loading control. Bar represents mean ± SD, n = 3 independent experiments. **P < 0.01, ***P < 0.001 compared with the control group. ^###^*P* < 0.001 compared with the PTF-α group. ^&&&^*P* < 0.001 compared with the ISO 6 h group.

In previous study, we found that ischemic preconditioning (IPC), a short-time (30 minutes) of OGD that do not affect the cell viability of neuron, induced ischemic tolerance against subsequent lethal OGD (4 hours) in neurons^25^,^26^. We then examined TIGAR expression at different time points after IPC. The results showed that TIGAR began to increase 1 h after IPC and reached peak at 6 h. (Fig. 1A, *P* < 0.05, *P* < 0.001 compared with the control group). The neurons were also exposed to 2% isoflurane for 30 minutes in an airtight chamber to induce ISO^27^. ISO increased TIGAR expression at 6 h (Fig. 1B, *P* < 0.01 compared with the control group).

TIGAR is defined as a TP53-induced glycolysis and apoptosis regulator, but its upregulation during cerebral ischemia/reperfusion is largely TP53 independent^24^ and appears to be regulated by SP1 transcription factor^28^. Particularly, cerebral preconditioning could increase the level of SP1 to promote the transcription of its target genes^29^, while SP1 plays a pivotal role in the regulation of TIGAR promoter^30^. To clarify the signals contributing to TIGAR upregulation during preconditioning, we thus treated the neurons with TP53 inhibitor pifithrin-α(PTF-α)^31^ and SP1 inhibitor mithramycin A^32^ before preconditioning. ISO markedly increased TIGAR at 6 h after preconditioning and so did PTF-α groups(Fig. 1C, *P* < 0.001 compared with the control group), whereas pretreatment with mithramycin A remarkably reduced the expression of TIGAR (Fig. 1C, *P* < 0.001 compared with the ISO6h), suggesting that SP1 might mediate TIGAR upregulation during preconditioning.

**Figure 2:**
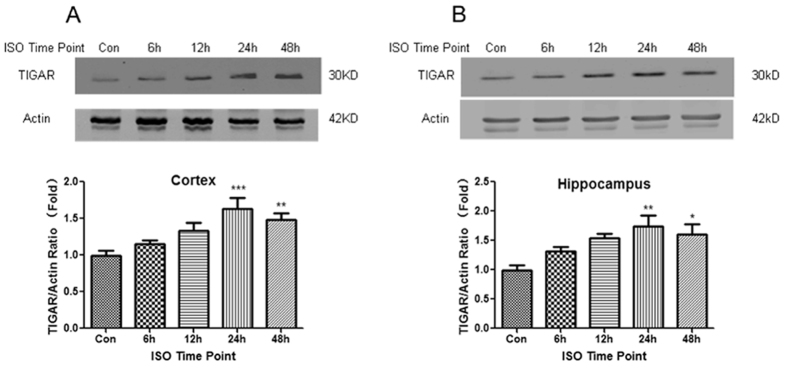
Isoflurane preconditioning (ISO) upregulats TIGAR in mouse brain. Mice were exposed to 1% isoflurane for 3 h to induce ISO. Cortex (**A**) and hippocampus (**B**) were dissected 6, 12, 24 and 48 h after ISO. The protein levels of TIGAR were detected with Western blot analysis. β-actin levels were used as loading control. Bar represents mean ± SD, n = 4. *P < 0.05, **P < 0.01, ***P < 0.001 compared with control group.

We also examined TIGAR expression in a mouse ISO model. The TIGAR expression in cortex and hippocampus barely had no change in the first few hours after ISO, while it was significantly upregulated at 24 h after ISO and maintained for another 24 h (Fig. 2A,B, *P* < 0.05, *P* < 0.01, *P* < 0.001 compared with the control group).

All these results suggest that ischemic preconditioning and isoflurane preconditioning both unregulated TIGAR in cortical neurons through SP1 transcription factor.

### TIGAR contributes to ischemic tolerance induced by preconditioning *in vitro* and *in vivo*

**Figure 3:**
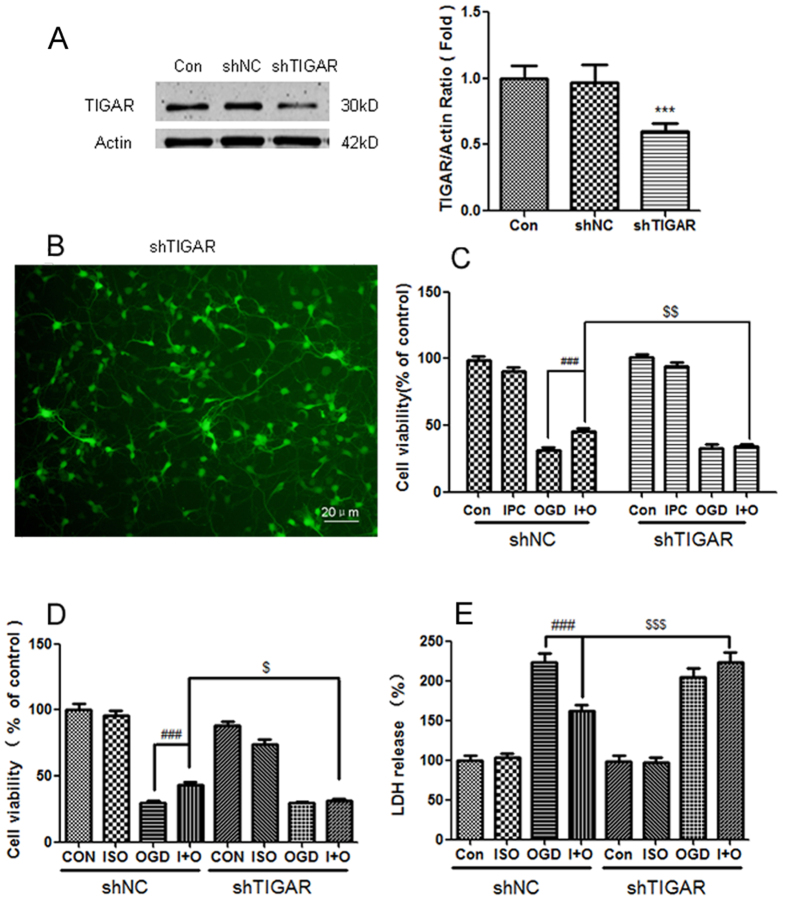
TIGAR contributes to ischemic tolerance induced by isoflurane preconditioning (ISO) and ischemic preconditioning (IPC) in cortical neurons. (**A**,**B**) The neurons were infected with LV-sh-TIGAR or LV-sh-scramble (negative control, NC). The efficiency of TIGAR knockdown was evaluated with Western blotting (**A**) and GFP fluorescence (**B**). (**C**) TIGAR knockdown cancelled IPC-induced neuroprotection. The neurons were subjected to OGD for 4 h at 24 h after IPC treatment. The cell viability was detected with CCK-8 assay. (**D**,**E**) TIGAR knockdown cancelled ISO-induced neuroprotection. The neurons were subjected to OGD for 4 h at 24 h after ISO treatment. The cell viability was detected with CCK-8 (**D**) and LDH (**E**) assay. Bar represents mean ± SD, n = 3 independent experiments. ***P < 0.001 compared with the control group. ^###^*P* < 0.001 compared with the OGD group. ^$^*P* < 0.05, ^$$^*P* < 0.01, ^$$$^*P* < 0.001 compared with the ISO + OGD or IPC +  OGD group.

To determine whether TIGAR contributes to ischemic tolerance induced by preconditioning, we transfected the cortical neurons with LV-shTIGAR or LV-sh-scramble (LV-shNC). After transfection, 78.2% of neurons in transfected group displayed GFP fluorescence, and TIGAR expression in the neurons infected with LV-shTIGAR was only 0.54 of that in LV-shNC group (Fig. 3A,B, *P* < 0.001). Then the transfected neurons were subjected to preconditioning stimuli and OGD treatment. OGD of 4 h induced serious cell damage in neurons, while IPC significantly attenuated the neuronal injury caused by OGD (Fig. 3C, *P* < 0.001 compared with OGD group). However, transfection with LV-shTIGAR markedly eliminated IPC’s protection against OGD injury in cortical neurons (*P* < 0.01 compared with LV-shNC + IPC + OGD). ISO also remarkably attenuated the neuronal injury induced by OGD as indicated by CCK8 assay (Fig. 3D, *P* < 0.001 compared with OGD), while ISO elicited neuroprotection against OGD was eliminated with TIGAR knockdown in cortical neurons (*P* < 0.05 compared with LV-shNC + ISO + OGD). Apoptosis or necrosis induced cell membrane destruction, resulting in the release of enzymes from the cytoplasm to the medium. The lactate dehydrogenase (LDH) assay was often used to test cytotoxicity in neurons. Similar to the results of CCK-8, ISO + OGD group showed lower release of LDH than OGD group (Fig. 3E, *P* < 0.001), but TIGAR knockdown significantly elevated LDH leakage from neurons (*P* < 0.001 compared with LV-shNC + ISO + OGD).

**Figure 4:**
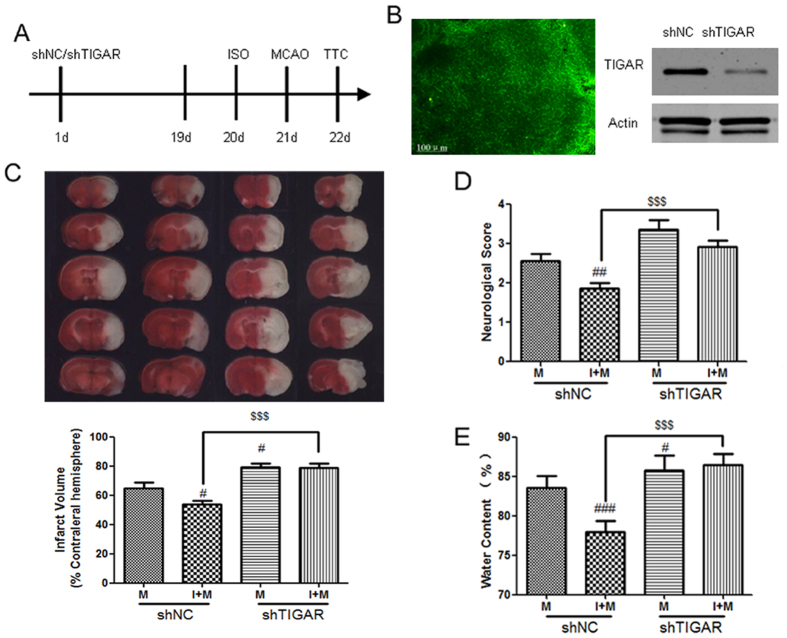
TIGAR contributes to ischemic tolerance induced by ISO *in vivo*. (**A**) The protocols of the *in vivo* experiments. Mice were injected with LV-shTIGAR or LV-shNC at the right lateral ventricle and the striatum (2 μL per site; 5 × 10^8^ TU). TIGAR expression in cortex were evaluated at the 19th day. The mice was subjected to ISO (the 20th day) and MCAO/reperfusion (the 21th day). (**B**)The efficiency of TIGAR knockdown in cortex was evaluated by GRP immunoflurescene and TIGAR immunoblotting. (**C**) TTC staining of brain sections showed that TIGAR knockdown increased the infarct volume. Infarct brain regions display white after TTC staining. (**D**) TIGAR knockdown aggravated the neurological deficits. (**E**) TIGAR knockdown aggravated brain edema. Bar represents mean ± SD, n = 6. ^#^*P* < 0.05, ^##^*P* < 0.01, ^###^*P* < 0.001 compared with the MCAO group. ^$$$^*P* < 0.001 compared with the LV-shNC + ISO + MCAO group.

To further verity TIGAR’s contribution to preconditioning *in vivo*, the lentivirus encoding shTIGAR or shNC was injected into right lateral ventricle and striatum. Nineteen days later, the lentivirus mediated TIGAR knockdown in mouse brain was verified by GFP immunofluorescence and Western blot (Fig. 4A,B). Then the middle cerebral artery was occluded for 2 hours and reperfused for 24 hours. In the mice treated with LV-shNC, ISO + MCAO significantly reduced infarct volumes, improved neurological deficits and reduced brain edema (Fig. 4C–E, *P* < 0.01, *P* < 0.001 compared with the MCAO group). However, in the mice treated with LV-shTIGAR, LV-shTIGAR + ISO + MCAO significant aggravated the infarct volume, neurological deficits and brain edema (*P* < 0.001 compared with LV-shNC + ISO + MCAO group), suggesting that TIGAR knockdown also abolished ischemic tolerance induced by ISO *in vivo*.

All these results suggest that TIGAR contributes to the neuroprotection induced by cerebral preconditioning *in vivo* and *in vitro*.

### TIGAR promotes NADPH and GSH production to scavenge ROS in cortical neurons during preconditioning

**Figure 5:**
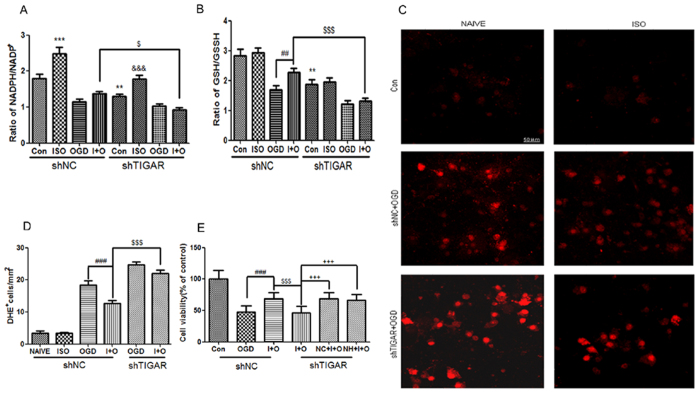
TIGAR promotes NADPH and GSH production and ROS clearance during preconditioning in cortical neurons. The neurons were infected with LV-sh-TIGAR or LV-shNC at DIV2. Neurons were subjected to OGD for 4 h at 24 h after IPC treatment. Cellular NADPH and GSH was evaluated at 3 h after reperfusion. (**A**) The level of NADPH/NADP^+^ in neurons. (**B**) The level of GSH/GSSG in neurons. (**C**) Representative photographs and quantification graph (**D**) of DHE fluorescence showed the levels of ROS in neurons at 3 h after reperfusion. Scale bar = 50 μm. (**E**) NAC and NADPH rescued neurons from TIGAR knockdown. Neurons were incubated with NAC (NC) 100 μM or NADPH (NH) 10 μM 30 min before preconditioning and then subjected to ISO and OGD/reperfusion. Bar represents mean ± SD, n = 3 independent experiments. **P < 0.01, ***P < 0.001 compared with the control group. ^##^*P* < 0.01, ^###^*P* < 0.001 compared with the OGD group. ^$^*P* < 0.05, ^$$$^*P* < 0.001 compared with the ISO + OGD group. ^&&&^*P* < 0.001 compared with NC + ISO. ^+++^*P* < 0.001 compared with LV-shTIGAR + ISO + OGD.

We then examined whether TIGAR is involved in NADPH production and ROS clearance during preconditioning. The results showed that the production of NADPH increased after ISO in cortical neurons (Fig. 5A, *P* < 0.001 compared with control), while OGD/reperfusion for 3 h consumed amount of NADPH with or without ISO. TIGAR knockdown significantly reduced NADPH production induced by ISO (*P* < 0.001 compared with NC + ISO, *P* < 0.05 compared with LV-shNC + ISO + OGD). Unlike NADPH, the level of GSH did not change after ISO, but ISO +OGD group maintained a high level of GSH compared with OGD group (Fig. 5B, *P* < 0.01), indicating that ISO alleviates GSH consumption in neurons subjected to OGD. When TIGAR was suppressed with LV-shTIGAR, the level of GSH in neurons significantly decreased (*P* < 0.01 compared with LV-shNC + CON). Importantly, compared with LV-shNC + ISO + OGD, LV-shTIGAR + ISO + OGD increased GSH consumption (*P* < 0.001). We also examined the intracellular ROS level with DHE staining. The naive and ISO group basically had little positive cells with DHE staining, but the red fluorescence in OGD group was very strong. ISO + OGD treatment limited the formation of ROS (Fig. 5C,D, *P* < 0.001 compared with OGD), while LV-shTIGAR + ISO + OGD clearly increased the DHE staining compared with LV-shNC + ISO + OGD group (*P* < 0.001).

N-acetyl-L-cysteine (NAC) is an effective antioxidant that helps to increase GSH synthesis and directly scavenges ROS^33^. We then investigated if NAC or NADPH could rescue the neurons from TIGAR deficiency after ISO and OGD treatment. The results showed that LV-shTIGAR abrogated the ischemic tolerance induced by ISO (Fig. 5E, *P* < 0.001 compared with LV-NC + ISO + OGD), while supplementation of NAC or NADPH both significantly attenuated the neuronal injury caused by TIGAR knockdown (*P* < 0.001 compared with LV-shTIGAR + ISO + OGD), indicating that inhibition of PPP flux and ROS production might contribute to neuronal injury induced by TIGAR knockdown.

**Figure 6:**
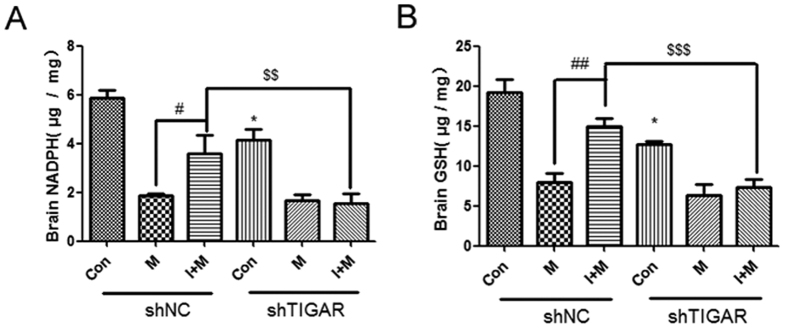
TIGAR increases NADPH production and ROS clearance in cortex during preconditioning. TIGAR knockdown and ISO/MCAO model was as described in Fig. 4A. The NADPH and GSH in cortex was evaluated at 3 h after MCAO/reperfusion. (**A**) The level of NADPH in brain. (**B**) The level of GSH in brain. Bar represents mean ± SD, n = 5. *P < 0.05 compared with the control group. ^#^*P* < 0.05, ^##^*P* < 0.01 compared with the MCAO group. ^$$^*P* < 0.01, ^$$$^*P* < 0.001 compared with the ISO + MCAO group.

We also determined the NADPH and GSH production in the mouse brain after TIGAR knockdown and ISO treatment. Similar to the *in vitro* results, ISO + MCAO treatment helped reduce consumption of NADPH and GSH compared with MCAO (Fig. 6A,B, *P* < 0.05), but LV-shTIGAR + ISO + MCAO significantly decreased the levels of NADPH and GSH in the cortex (*P* < 0.01 or *P* < 0.001 compared with the LV-shNC + ISO + MCAO group), indicating that TIGAR is also involved in NADPH and GSH production induced by ISO *in vivo*.

All these results indicate that TIGAR promotes the production of NADPH and GSH to scavenge ROS during preconditioning.

### TIGAR contributes to anti-apoptotic effects during preconditioning in cortical neurons

**Figure 7:**
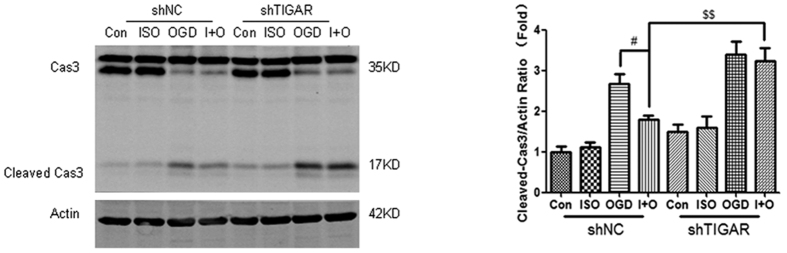
TIGAR contributes to ISO- induced anti-apoptotic effects in cortical neurons. The neurons were infected with LV-sh-TIGAR or LV-shNC at DIV2. The neurons were subjected to OGD for 4 h at 24 h after IPC treatment. The cells were harvested at 3 h after reperfusion and caspase-3 was measured by Western blot analysis. β-actin levels were used as the loading control. Bar represents mean ± SD, n = 3 independent experiments. ^#^*P* < 0.05 compared with the OGD group. ^$$^*P* < 0.01 compared with the ISO + OGD group.

TIGAR is demonstrated to reduce ROS dependent apoptosis through PPP flux. We thus examined whether TIGAR shows anti-apoptotic effects during preconditioning. Bcl-2 and Bax proteins play important roles in mitochondrial pathway of apoptosis, while caspase3 is the key executor of apoptosis^34^. ISO markedly increased Bcl-2 expression (Supplementary Fig. S1, *P* < 0.01 compared with control), while TIGAR knockdown reduced Bcl-2 upregulation induced by ISO (*P* < 0.05 compared with ISO). Although there is no dramatic change of Bcl-2 and Bax between OGD and ISO + OGD groups (*P* > 0.05), LV-shTIGAR + ISO + OGD markedly reduced Bcl – 2 and increased Bax level (*P* < 0.05 or *P* < 0.01 compared to LV-NC + ISO + OGD). Consistently, OGD/reperfusion for 3 h increased caspase3 cleavage, while ISO + OGD prevented caspase-3 activation (Fig. 7, *P* < 0.05 compared with OGD). However, LV-shTIGAR + ISO + OGD increased caspase3 cleavage compared with LV-shNC + ISO + OGD (*P* < 0.01). These results suggest that TIGAR contributes to anti-apoptotic effects during preconditioning.

## Discussion

**Figure 8:**
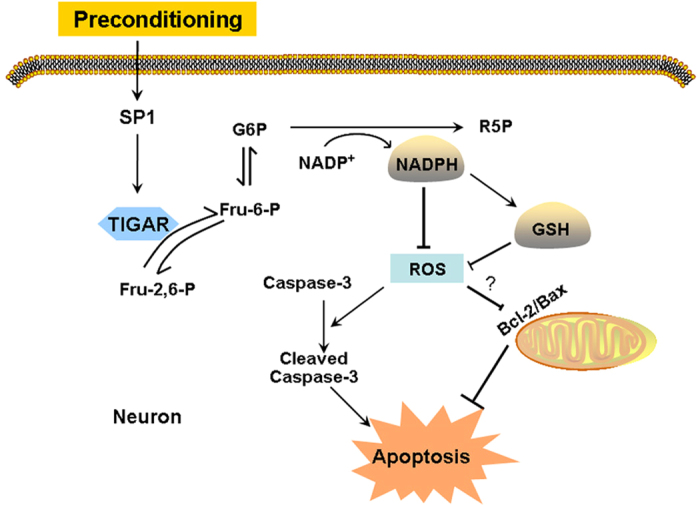
Proposed mechanisms by which TIGAR mediates cerebral preconditioning. Cerebral preconditioning upregulates TIGAR in neurons. TIGAR functions to increase fructose-6-phosphtae (Fru-6-P) and glucose-6-phosphate (G6P), resulting in production of NADPH and ribose-5-phosphate (R5P). TIGAR then helps ROS clearance through increased production of NADPH and GSH. Thus preconditioning prevents neuronal apoptosis induced by subsequent ischemia through inhibition of caspase-3 cleavage.

Cerebral preconditioning that induces neuronal tolerance to a subsequent lethal ischemia might bring insight in the treatment of ischemic stroke^4^,^5^,^35^. In this study, we used two cerebral preconditioning stimuli to explore the role of TIGAR in preconditioning. The results showed that both ischemic preconditioning (IPC) and isoflurane preconditioning (ISO) increased the expression of TIGAR *in vivo* and *in vitro*. IPC and ISO attenuated the ischemic neuronal injury, while knockdown of TIGAR abolished the neuroprotection induced by cerebral preconditioning. Preconditioning promoted PPP flux, as evidenced by increased NADPH and GSH, which helped to reduce ROS formation and neuronal apoptosis during subsequent ischemia, while TIGAR knockdown cancelled these effects. These results suggest that TIGAR plays an important role in cerebral preconditioning through production of NADPH from PPP flux, scavenging of ROS and inhibition of apoptosis. (Fig. 8).

TIGAR is a novel target protein involved in glucose metabolism and cerebral ischemic injury^18^,^19^,^24^,^36^. The previous study showed that TIGAR increases PPP flux and promotes the production of NADPH to remove reactive oxygen species (ROS) and to maintain cell survival^24^,^37^,^38^,^39^. TIGAR also protects ischemic brain injury via enhancing PPP flux and preserving mitochondria function^24^. We thus hypothesize that TIGAR might be involved in cerebral preconditioning. Our data showed increased TIGAR levels in primary cultured cortical neurons after preconditioning. Preconditioning might upregulate TIGAR through SP1 transcription factor. Both IPC and ISO protected against OGD injury, while pretreatment with LV-shTIGAR blocks preconditioning-induced tolerance in primary neurons. In agreement with the *in vitro* data, in mice exposed to isoflurane, TIGAR is increased in cortex and hippocampus. The protective effect of ISO against MCAO was eliminated with TIGAR knockdown in brain. We thus conclude that TIGAR contributes to cerebral preconditioning and protects the neuron against ischemic injury.

Reactive oxygen species (ROS) play important roles in regulation of cell apoptosis and homeostasis during brain ischemia^16^. It has been demonstrated that ischemia/reperfusion increases the formation of ROS, imposing oxidative stress on proteins, DNA, lipids, and organelles in cells^40^,^41^. Glutathione(GSH), the main source of mercapto in a vast majority of living cells, plays a vital role in maintaining redox state of proper protein thiol, while NADPH may maintain the level of reduced form of glutathione^18^,^42^. TIGAR could inhibit glycolysis and increase the pentose phosphate pathway (PPP) as compensation, resulting in increased NADPH production, which help limit reactive oxygen species (ROS)^37^,^43^,^44^. Previous finding showed that ischemia/reperfusion increases mitochondrial localization of TIGAR, which preserves mitochondria function and produces more NADPH and GSH to alleviates cellular oxidative stress damage^24^, and exogenous NADPH significantly protects neurons against ischemia/reperfusion^45^. Consistently with these results, our data showed that isoflurane preconditioning reduced the NADPH and GSH consumption and ROS accumulation in mouse brain and cortical neurons. Knockdown of TIGAR abolished preconditioning-induced production of NADPH and GSH and ischemic tolerance, while supplementation of ROS scavenger NAC and PPP product NADPH effectively rescue the neuronal injury caused by TIGAR deficiency. These results suggest that NADPH and GSH mediated ROS clearance is essential for TIGAR’s contribution to ischemic tolerance during preconditioning.

Apoptosis has long been known to take part in the ischemia/reperfusion induced neuronal death, whereas preconditioning protects brain tissues from ischemia/reperfusion by suppression of apoptotic pathways^4^,^5^,^35^. TIGAR is shown to reduce ROS dependent apoptosis through PPP flux. In TIGAR-deficient cells, the level of ROS-dependent apoptosis increases^43^. Consistent with these results, our data showed that preconditioning inhibited OGD/reperfusion-induced caspase-3 activation in neurons, while knockdown of TIGAR increased caspase-3 cleavage. These results implied that TIGAR knockdown could cancel preconditioning induced anti-apoptotic effects.

ROS is the key mediator of cellular oxidative stress and neuronal apoptosis during cerebral ischemia^46^. Bcl-2 family of proteins are involved in regulation of mitochondria-dependent pathway for apoptosis^34^. The anti-apoptotic protein Bcl-2 inhibits apoptosis by preventing the mitochondrial membrane depolarization and inhibiting caspase-3 activation, whereas the pro-apoptotic protein Bax promotes apoptosis by inducing mitochondrial membrane depolarization^46^. Evidences showed that Bcl-2 is implicated in the preconditioning-induced ischemic tolerance^47^,^48^, and isoflurane preconditioning increases Bcl-2 expression to block cytochrome c release from mitochondria and to inhibit neuronal apoptosis^49^. In addition, Bcl-2 is a complex mediator involved in neuronal apoptosis regulated by ROS. Some studies proposed that Bcl-2/Bcl-xL may function to prevent the generation of ROS during apoptosis^50^. Enhanced oxidative stress and susceptibility to oxidants are evident in the brains of Bcl-2 deficient mice compared to wild type mice^51^. Bcl-2 also protects cells from oxidative stress-induced death and suppresses lipid peroxidation^52^. However, mounting evidence also showed that ROS may induce apoptosis by regulating Bcl-2/Bax dependent mitochondrial apoptosis pathway. ROS sensitizes T cell apoptosis by decreasing expression of Bcl-2^53^. ROS regulates phosphorylation, ubiquitination or cysterine oxidation of Bcl-2, resulting in decreased antiapoptotic Bcl-2 expression and increased proapoptotic Bax expression^54^,^55^. In the present study, we showed that TIGAR deficiency caused the rise of ROS level and the downregulation of Bcl-2/Bax under ISO + OGD treatment. However, from the current data, it has not been clear whether Bcl-2 proteins act as downstream or upstream of ROS formation during neuronal apoptosis under TIGAR deficiency. Further investigation is needed to determine the relationship between Bcl-2 and ROS following TIGAR depletion.

In summary, our results showed that TIGAR participates in the cerebral preconditioning through clearance of ROS and inhibition of neuronal apoptosis. The combination of TIGAR and cerebral preconditioning may be a new therapeutic target for stroke prevention and treatment. Many findings from stroke are generalized to other neurodegenerative diseases. Thus this study might also broaden the possible therapeutic targets for a multiplicity of neurodegenerative diseases^56^,^57^.

## Methods

### Cell culture

Primary cultured cortical neurons was derived from the cortex of E17 embryonic mice (E15-17, Center for Experimental Animals of Soochow University, certificate No 20020008, Grade II). The national guidelines for laboratory animal care and use were followed in all animal experiments. Care and handling of animals were approved by the Ethical Committee of Soochow University. The cortices were dissected out into pre-cooled PBS, and incubated with 2.5% trypsin at 37 °C for 15 min. Then the sediment were mixed with 10 mL DMEM (Gibco) containing 10% fetal bovine serum and DNase I (Sangon Biotech) for 3 min. The sediment were homogenized by piepetting up and down and centrifuged at 500 g for 5 min. The pellets were suspended in neurobasal medium (NBM, Gibco) containing 10% B27 (Gibco) and 25 μM glutamate. The cell suspensions solution was passed through a 40 μm strainer. Then the desired number of cells was plated onto poly D-lysine (Sigma)-coated plates. The medium were replaced by NBM containing 10% B27, 0.5 μM glutamine after 24 hours. Half medium was changed every 2 days. Usually, the cortical neurons were used for experiment after 7 ~ 9 days.

### Lentivirus-mediated TIGAR knockdown in neurons

U6-EGFP-IRES-Puro (5 × 10^8 ^TU/ml, Gene ID 308589, NM 001012066) lentivirus was constructed by GENECHEM (Shanghai, China). At 2 days *in vitro* (DIV2), the medium were changed with NBM containing 10% B27, 0.5 μM glutamine and LV-shTIGAR (MOI = 10) or LV-sh-scramble (LV-sh-negative control, LV-shNC) for 24 h, and then changed back to the regular medium. Twenty-four hours after transfection, GFP expression fluorescence was observed and the GFP-positive cells were counted from five randomly chosen fields to determine transfection efficiency.

### *In vitro* isoflurane preconditioning (ISO), ischemic preconditioning (IPC) and oxygen glucose deprivation (OGD) models

After 7 days in culture, the neurons were exposed to 2% isoflurane (in 70% nitrogen and 30% oxygen) at 37 °C for 30 min in an airtight chamber. Neurons were then removed from the chamber and cultured under normal conditions. The duration and concentration of ISO exposure did not induce significant neuronal toxicity^27^.

For OGD model, the neurons were rinsed three times with Hepes balanced salt solution(HBSS:140 mM NaCl, 3.5 mM KCl, 12 mM MgSO_4_, 5 mM MaHCO_3_, 1.7 mM CaCl_2_, 0.4 mM kH_2_PO_4_, 10 mM Hepes, pH 7.3 ) and placed in a chamber (Billups-Rothenberg MC-101) which filled with 95% N_2_ and 5% CO_2_ at 37 °C for 4 h. Neurons were then removed from the anaerobic chamber, and replaced with the normal culture medium and cultured under normal conditions. For IPC model, the neurons were deprived of oxygen and glucose for 30 min, an insult that did not induce neuronal death^25^.

### Cell viability assay

Cell viability of cultured primary neurons was evaluated with Cell Counting Kit-8 (CCK-8, Dojindo Laboratories, Kumamoto, Japan) or lactate dehydrogenase (LDH) assay kit (Beyotime). For CCK8 assay, the neurons were seeded in 96-well cell culture plates. After 24 h after OGD/reperfusion, 100 μL medium and 10 μL CCK-8 were added per well and incubated with the cells at 37 °C for 2 h. The optical densities were measured at 450 nm with a microplate reader (ELX 800, Bio-Tek). For LDH assay, the neurons and culture medium were lysed in PBS containing 1% Triton X-100 at 37 °C for 30 min, respectively. The LDH activities in both the cell lysates and the culture mediums were assayed with the assay kit following the manufacturer’s instructions. LDH leakage was calculated as follows: LDH leakage (%) = LDH culture medium/(LDH culture medium + LDH cell lysates) × 100%. Every experiment was repeated three times.

### Isoflurane preconditioning (ISO) and middle cerebral artery occlusion (MCAO) model in mice

Male ICR mice (24–26 g; Center for Experimental Animals of Soochow University, certificate No. 20020008, Grade II) were exposed to 1% isoflurane (in 70% nitrogen and 30% oxygen) for 3 h in an airtight chamber. Then the mice were allowed to recover in their original cages. Control mice were placed in the airtight chamber for 3 h with pure air (70% nitrogen and 30% oxygen)^27^.

For MCAO model, mice were anesthetized with intraperitoneal injection of 4% chloral hydrate. The MCAO was produced by intraluminal occlusion of the right MCA using a silicone-coated nylon (6-0) monofilament (Doccol Corporation 602356PK5Re). After 2 h of occlusion, filament was withdrawn to allow blood reperfusion. Cerebral blood flow was monitored with LDF ML191 Laser Doppler Blood Flow Meter. The mice were placed on a heating pad to maintain rectal temperature at 37.0 ± 0.5 °C during the surgery until recovery from anesthesia.

### Evaluation of neurological score, infarct volume and brain water content

After reperfusion for 24 h, the neurological deficits were evaluated by an investigator blinded to the experimental treatments^58^. The following scale rating was used: 0, normal motor function; 1, flexion of torso and contralateral forelimb when mouse is lifted by the tail; 2, circling to the contralateral side when mouse is held by the tail on a flat surface but normal posture at rest; 3, leaning to the contralateral side at rest; 4, no spontaneous motor activity. Then the mice were sacrificed and the brain was cut into five slices. Brain sections were stained with 1% 2, 3, 5- triphenyltetrazolium chloride (TTC) (Sinopharm Chemical Reagent Co.,Ltd) solution at 37 °C for 15 min. The TTC-stained brain slices were photographed with a digital camera and the infarct sizes were measured using Image J. Considering the infarct volume expansion due to edematous change, the infarct volume was calculated with the following formula: infarct volume =  (red area of contralateral side – red area of ipsilateral side)/contralateral hemisphere ×100%^59^. After TTC staining, the wet weight of the brains was quantified. Then these brains were desiccated at 105 °C for 48 h until the weight was constant and the total weight of the dried TTC-stained brains was obtained. The water content of each brain was measured as follows: water content = (wet weight − dried weight)/wet weight × 100%^60^.

### Intracerebral injection of lentivirus

For lentivirus injection, the mice were placed on a stereotaxic apparatus (Stoelting). Then the lentivirus encoding shTIGAR (LV-shTIGAR) or LV-sh-scramble (LV-shNC) (2 μL per site, 5 × 10^8 ^TU/ml) were injected into lateral ventricle(0.4 mm anterior to the bregma, 0.8 mm lateral, 2.5 mm deep) and striatum (0.4 mm anterior to the bregma, 1.8 mm lateral, 3.5 mm deep). The efficiency of TIGAR knockdown was evaluated with the GFP fluorescence and Western blot analysis in the cortex. Twenty-one days later, ISO and/or MCAO/reperfusion models were performed in these animals.

### Measurement of NADPH and GSH levels

The neuronal NADP^+^, NADPH, GSH and glutathione disulfide (GSSG) were measured 3 h after reperfusion with the EnzyChrom NADP^+^/NADPH assay kit (BioAssay Systems ECNP-100) and GSH Kit (Byeotime S0053) following manufacturer’s instructions. The data were expressed as the ratio of NADP^+^/NADPH and GSH/GSSG. Three hours after MCAO/reperfusion and/or ISO, the cortex was homogenated in PBS. Then NADPH and GSH were measured with the EnzyChrom NADPH/NADP^+^ assay kit and Mouse GSH ELISA kit (Xinle xl-Em1124).

### Drug treatment

Cortical neurons were pretreated with mithramycin A^32^ (Sangon Biotech M0668) 300 nM or pifithrin-α^31^ (Sigma P4236) 10 μM 24 h before preconditioning. Neurons were pretreated with NADPH^45^ (Beyotime ST360) 10 μM or N-acetyl-L-cysteine^33^ (NAC, Sigma A9165) 100 μM 30 min before preconditioning.

### DHE fluorescence

The neurons were treated with the PBS containing dihydroethidium (DHE, 2 μM; Sigma) for 30 min at 37 °C. After rinsed in PBS, the DHE fluorescence was observed with a fluorescence microscope (Olympus). Positive cells marked with DHE staining per mm^2^ were counted in each group.

### Western blot analysis

The cortical neurons were rinsed twice with cooled PBS and lysed in a buffer containing Tris-HCl (PH 7.4) 10 mM, NaCl 150 mM, 1% Triton X-100, 1% sodium dexoxycholate, 0.1% SDS, edetic acid 5 mM, and 1 protease inhibitor cocktail tablet (Roche)/10 ml. The ipsilateral cortex and hippocampus were homogenized and proteins were also extracted with this lysis buffer. Equal amounts (10–30 μg) of total protein extracts were separated by SDS-PAGE and transferred to nitrocellulose membranes. The membranes were incubated with primary antibodies against rabbit anti-TIGAR(1:1000; Abcam 37910), rabbit anti-Bcl-2 (1:1000; Santa cruz SC-492), rabbit-anti-Bax (1:200; Cell signaling 2772), rabbit anti-Caspase-3 (1:1000; Cell signaling 9665), and mouse anti-β-actin (1:10000; Sigma A5441) at 4 °C overnight. After rinsed in the TBST containing 0.05% Tween 20, the membranes were incubated with secondary antibodies (1:20000; LI-COR Biosciences, anti-Mouse 926–32212; anti-Rabbit 926–32213) at room temperature for 2 hours. The membranes were revealed by Odyssey Two-Color Infrared Imaging Systen (LI-COR, Linclon, NE, USA) following the manufacturer’s instructions. The proteins were analyzed with Image J and normalized to the loading control (β-actin).

### Statistical analysis

Data were expressed as mean ± SD. Significant differences between groups were determined with one-way ANOVA. Post-hoc analysis was carried out with the Newman-Keuls test.

## Additional Information

**How to cite this article**: Zhou, J.-H. *et al.* TIGAR contributes to ischemic tolerance induced by cerebral preconditioning through scavenging of reactive oxygen species and inhibition of apoptosis. *Sci. Rep.* **6**, 27096; doi: 10.1038/srep27096 (2016).

## Supplementary Material

Supplementary Information
