# Supplementary material for: TIGAR contributes to ischemic tolerance induced by cerebral preconditioning through scavenging of reactive oxygen species and inhibition of apoptosis
Source: Sci Rep. 2016 Jun 3;6:27096. doi: 10.1038/srep27096 (PMC4891774; doi:10.1038/srep27096)

Supplementary Information

TIGAR contributes to ischemic tolerance induced by cerebral preconditioning through scavenging of reactive oxygen species and inhibition of apoptosis

Jun-Hao Zhou[1], Tong-Tong Zhang, Dan-Dan Song, Yun-Fei Xia, Zheng-Hong Qin, and Rui Sheng*

[1]Department of Pharmacology and Laboratory of Aging and Nervous Diseases, Soochow University School of Pharmaceutical Science, Suzhou, China

*Corresponding author:
Rui Sheng, PhD
Department of Pharmacology and Laboratory of Aging and Nervous Diseases,
Soochow University School of Pharmaceutical Science, Suzhou, China
199 Ren Ai Road, Suzhou 215123
Phone Number: 86-512-65882071
Fax Number: 86-512-65882071
Email: sheng_rui@163.com

Supplementary Figure 1. TIGAR deficiency caused the downregulation of Bcl-2/Bax under ISO+OGD treatment. The neurons were infected with LV-sh-TIGAR or LV-shNC at DIV2. The neurons were subjected to OGD for 4 h at 24 h after IPC treatment. The cells were harvested at 3h after reperfusion and subjected to Western blot analysis. (A) TIGAR knockdown reduced the expression of Bcl-2. (B) TIGAR knockdown increased the expression of Bax. Bar represents mean ±SD, n=3 independent experiments. ** *P*< 0.01 compared with the control group. $ *P* <0.05, $$ *P* < 0.01 compared with the ISO+OGD group, & *P* < 0.05 compared with NC+ISO.

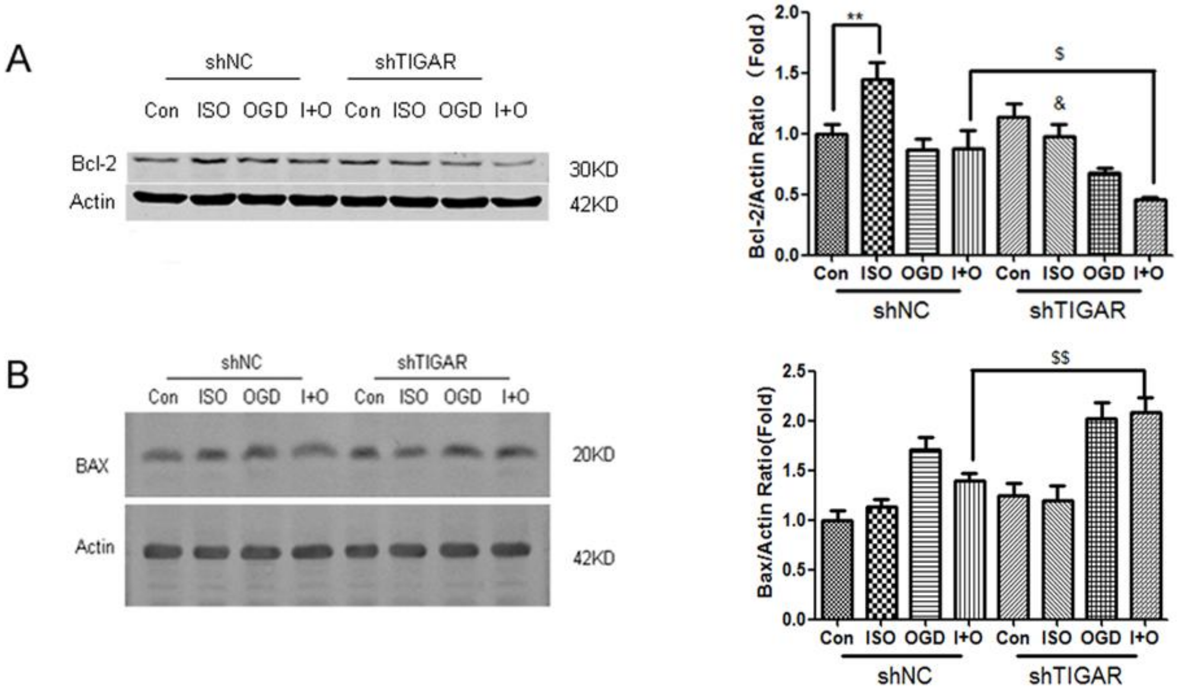

Supplement: Supplementary Information [file srep27096-s1.pdf]
